# Comparison of the expression of 5 heat shock proteins in benign and malignant salivary gland tumor tissues

**DOI:** 10.3892/ol.2013.1166

**Published:** 2013-01-30

**Authors:** GUILAN WANG, XIAOLIN GU, LI CHEN, YINMEI WANG, BIN CAO, QUN E

**Affiliations:** 1 Department of Pathological Anatomy, Medical College, Nantong University, Nantong, P.R. China; 2 Departments of Stomatology, The First People’s Hospital of Nantong, Nantong, P.R. China; 3 Otolaryngology (ENT), The First People’s Hospital of Nantong, Nantong, P.R. China; 4 Department of Pathology, People’s Hospital of Haian County, Nantong, P.R. China

**Keywords:** heat shock proteins, proliferating cell nuclear antigen, salivary gland tumor, significance

## Abstract

The objective of this study was to analyze the significance and potential value of heat shock proteins (HSPs) in salivary gland tumors. We found that expression of HSP60, HSP70, HSP86 and HSP84 were all upregulated in both salivary gland benign tumors and malignant tumors, and that the expression of HSP70, HSP86 and HSP84 was more greatly overexpressed in the malignant tumors (each P<0.01). For HSP27, expression was upregulated both in malignant and benign tumors, with less expression observed in malignant tumors (P<0.01). In malignant tumors, expression of HSP27 was negatively correlated with the age of the patients, size of the tumor tissue, occurrence of neural invasion and metastasis (each P<0.05). Additionally, in malignant tumors, HSP70 and HSP86 were both positively correlated with occurrence site, neural invasion and metastasis (each P<0.05), while HSP60 was only negatively correlated with the age of the patients (P<0.05). HSP86 was also positively correlated with malignant degree (P<0.01). In malignant tumors, the proliferation index (PI), which was marked by proliferating cell nuclear antigen (PCNA; PCNA-PI) was 49.95±14.569, which was significantly higher compared with that in benign tumors (P<0.001), which was in accordance with the upregulation of HSP70, HSP86 or HSP84; however, an adverse correlation was found between HSP27 expression and PCNA (each P<0.05). In conclusion, these results suggest that HSPs are involved in the occurrence and development of salivary gland tumors. HSP70, HSP86 and HSP84 retained the higher multiplication capability of the malignant tumor cells, however, HSP27 did not. Thus, the upregulation of HSP70, HSP86 and HSP84 and the downregulation of HSP27 may all be used as biomarkers of the occurrence and development of malignant salivary gland tumors. Moreover, the extremely high expression of HSP86 and HSP84 in benign tumors indicates the malignant transformation potential.

## Introduction

The salivary gland is a major exocrine gland of the human body. Due to complexities in the occurrence sites, alveolar types and structural origins, the underlying mechanisms of salivary gland tumors may be significantly different. Moreover, salivary gland tumors are prone to recur after treatment and malignant transformation. For these reasons, identifying methods of diagnosis and prevention of salivary gland tumors remains important work for clinical physicians.

Heat shock proteins (HSPs) or stress proteins (SPs), are a series of important molecular chaperones, which are expressed in the cell membranes and cytoplasm as well as nuclei of both prokaryotic and eukaryotic organisms, which function to regulate the growth and proliferation of the cells (1,2). Based on the molecular weight and homology, HSPs are classified into HSP90, HSP70 (constitutive and inductive), small molecular weight HSPs and ubiquitin. Recently, HSPs have been found to be upregulated in numerous tumors varying with different causes, tissues or distribution, and interacting with other client proteins (1,3). Thus, it was confirmed that HSP expression is involved in the occurrence and development of a number of tumors as well as their biological behaviors, prognosis and treatment effect (4–6).

However, to date, few studies on HSPs in oral tumors, particularly in the salivary gland, have been conducted (7), and knowledge of their exact significances is limited.

In this study, the expression characteristics of the HSP family, including HSP27, HSP60, HSP70 and two subtypes of HSP90, HSP86 and HSP84, in salivary gland tumors, as well as their variance with gender, age, location, size, neural invasion, local metastasis and proliferation index (PI) in malignant tumor patients was studied. Also, the possible role and pathological significance of HSPs on the occurrence and development of salivary gland tumors was assessed.

## Materials and methods

### Specimens

In total, 81 cases of formalin-fixed, paraffin-embedded specimens of salivary gland tumors which had been surgically removed were collected between 1991 and 2009. Complete clinical and pathological records were investigated. Samples were obtained from the First People’s Hospital of Nantong, People’s Hospital of Haian County and Department of Pathology, Medical College of Nantong University, Nantong, China. Of the 81 cases, 10 had 1.5-cm peritoneal non-tumor salivary gland tissues. Our study was approved by the local medical Ethics Committee, and prior written informed consent and approval was obtained from the patients.

### Reagents

Mouse anti-human HSP27, HSP60 and HSP70, and the rabbit anti-human HSP86, HSP84 and HSP70 were all products from NeoMarkers. Immunohistochemical (IHC) high-sensitivity S-P kits and auxiliary reagents were purchased from Fuzhou Maixin Biotechnology Development Co., Ltd. The mouse anti-human proliferating cell nuclear antigen (PCNA) was purchased from Dako Co., Ltd. The Picture double staining kits (kit95-9999 and kit87-9999) were purchased from Zymed Co. The working concentrations of these primary antibodies were all set at 1:50.

### Methods

After the specimens were continuously sliced to 4 μm thick, a double-blind method was used to pathologically diagnose and classify tumors in line with the standards proposed by WHO (2005)(8). The single factor expression and co-expression of HSPs and PCNA were detected by the IHC S-P method (DAB staining) and the IHC double-labeled double staining method. For the procedure of IHC double-labeled double staining, the HSPs were detected by the IHC three-step method, then with the help of an enhancer, the PCNA was detected by the IHC two-step method. The routine IHC S-P and double staining experiments were all performed according to the reagent’s instructions, and their positive-standards were consistent. During the experiment, microwave antigen retrieval was used with pH 6.0 citrate buffer of 10 mM. Known positive slices of infiltrating breast cancer were applied as the positive control group and phosphate-buffered saline (PBS) was applied to substitute the primary antibody as the negative control group. In addition, 10 specimens from the marginal normal mucosa of tumor were used as normal controls.

### Positive standards

The positive staining for HSP27 and HSP60 was located in the cytoplasm of the tumor cells, the positive staining for HSP86 and HSP84 in the cytoplasm with nuclear staining and the positive staining for HSP70 was located both in the nucleus and the cytoplasm of tumor cells. The staining degree was scored as 0, 1, 2 and 3 corresponding to no staining, mild yellow, brownish yellow and tawny, respectively. Then, 5 visual fields (magnificaion, ×200) were selected at random in all slices, and the positive ratio of the tumor cells among the total tumor parenchymal cells in the field was calculated, based on the following criteria; <5% scored as 0, 5–30% as 1, 30–60% as 2 and ≥60% as 3. The products of the above 2 indices were used to determine the expression strength of the tumor tissues, which were ranked as: 0, negative (−); 1–3, weak expression (+); 4–6, medium expression (++); and 7–9, strong expression (+++).

The PCNA was located in nucleus. Five visual fields (magnification, ×200) were detected in each of the slice at random, and the expression rate of the tumor cells is presented as PI inferred from the ratio between the positive cells and the whole cells in the visual field.

### Statistical analysis

All data were analyzed using the Statistical Package for Social Science (SPSS, Inc., Chicago, IL, USA) version 17.0. The Chi-square test and the Spearman’s rank correlation test was used in data of the categorical variables, and the t-test was used for comparison of the mean. When the sample size (n) was ≥40 and the expected count (T) was between 1 and 5, or when the sample size was <40 and T was <1, the likelihood-ratio Chi-square test was used. Fisher’s exact test (two-sided) was only used in a four-fold table. In all tests, the significance level was set at 0.05.

## Results

### Clinical data

In total, 81 cases aged from between 13 and 83 years (mean, 49.3) were included in the study, of which 42 were male and 39 were female. The occurrence sites included the parotid gland (n=64), the submandibular gland (n=7) and other small glands (n=10). For the tumors, 41 were benign (25 mixed tumors, 13 adenolymphoma and 3 others) and 40 were malignant with diameters of the tumor tissue ranging from 1.5 to 6 cm [7 malignant mixed tumor and 18 adenoid cystic carcinoma (ACC), 9 mucus epidermoid cancer and 6 other malignant tumors].

### Expression of 5 HSPs in benign or malignant salivary gland tumors

**Figure 1:**
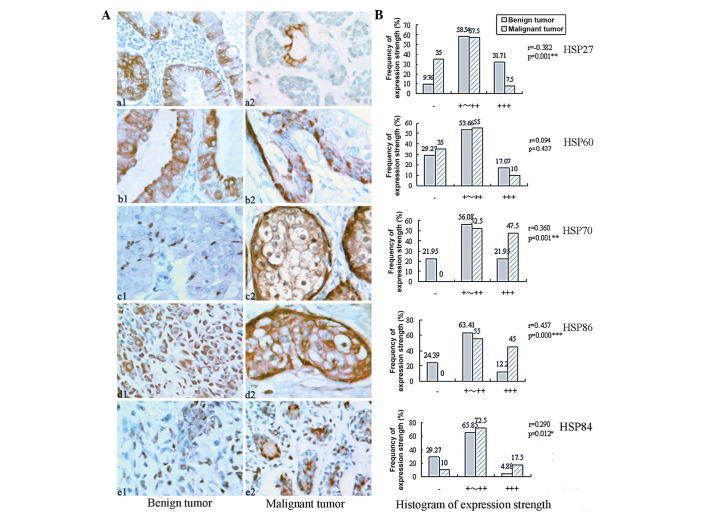
Expression location and strength of 5 HSPs in salivary gland tumors. (A) Expression location and strength of 5 HSPs (IHC-SP staining; magnification, ×400). HSP27 and HSP60 were located in the cytoplasm of the tumor epithelium, but in adenolymphoma they were mainly expressed in (a1) the lower epithelium and (b1) the top epithelium, respectively. HSP70 was significantly expressed in the nucleus and cytoplasm, but (d) HSP86 and (e) HSP84 were mainly located in the cytoplasm and partly expressed in nucleus. Expression of HSP27 was less in (a2) malignant tumor compared with (a1) benign tumor, but (c2) HSP70, (d2) HSP86 and (e2) HSP84 were all stronger in malignant tumor compared with (c1, d1 and e1) benign tumor. (B) Histograms of expression strength of the 5 HSPs. ^*^P<0.05; ^**^P<0.01; malignant tumor (group) vs. benign tumor (group). ^***^P<0.001. HSPs, heat shock proteins.

Five of the HSPs were positively expressed in normal salivary ducts, but negative in normal glands. Positive HSP27 and HSP60 were expressed in the cytoplasm of the tumor epithelium (Fig. 1Aa and b), but for adenolymphoma, HSP27 was mainly expressed in the lower epithelium (Fig. 1Aa1) and HSP60 in the upper (Fig. 1Ab1). HSP70 was expressed in the nucleus and cytoplasm (Fig. 1Ac). HSP86 and HSP84 were mainly expressed in the cytoplasm, with some in the nucleus (Fig. 1Ad and Ae). However, for the mixed tumors, they were mainly located in the cytoplasm of the gland ductal epithelium and the nucleus of myoepithelium.

The expression rates of HSP27, HSP60, HSP70, HSP86 and HSP84 were 90.24% (37/41), 70.73% (29/41), 78.05% (32/41), 75.61% (31/41) and 70.73% (29/41) in the benign tumor group, respectively. On the contrary, in the malignant tumor group, that of HSP27 and HSP60 both decreased to 65.00% (26/40), and those of HSP70, HSP86 and HSP84 increased to 100% (40/40), 100% (40/40) and 90% (36/40), respectively (P=0.006, 0.581, 0.002, 0.001 and 0.029, respectively). Moreover, compared with those in the benign tumors, the expression strength of HSP27 was lower in malignant tumor (r=−0.382, pr=0.001), but those of HSP70, HSP86 and HSP84 were higher (r= 0.360, 0.457 and 0.290, respectively; each P<0.01; Fig. 1B).

**Figure 2:**
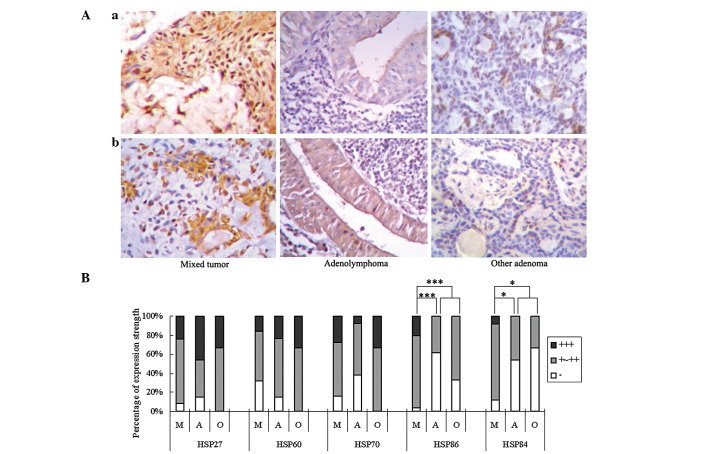
Expression comparison of 5 HSPs in benign types of salivary gland tumor (n=41). (A) Micrograph of expression strength of HSPs. (IHC-SP staining; magnification, ×400). Expression of (a) HSP86 and (b) HSP84 was greater in the mixed tumors, but less in the adenolymphoma and other benign tumor groups. (B) Histogram of expression strength of 5 HSPs. ^*^P<0.05; ^***^P<0.001. M, mixed tumor; A, adenolymphoma; O, other benign tumors; HSP, heat shock proteins.

In the three groups of benign tumors, the expression of HSP86 and HSP84 was greater in the mixed tumors, but less in the adenolymphoma group and other benign tumor group (Fig. 2A and B). For HSP86, the three positive grades accounted for 1, 19 and 5 cases, respectively, among the 25 mixed tumor cases; 8, 5 and 0, respectively, in the 13 adenolymphoma cases; and 1, 2 and 0 in the three other benign tumor cases (P<0.001). For HSP84, these figures were 3, 20 and 2, respectively, in the 25 mixed tumor cases; 7, 6 and 0, respectively, in the 13 adenolymphoma cases; and 2, 1 and 0 in the three other benign tumor cases (P<0.05). However, the expression of the 5 HSPs was not significantly different in the malignant types (each P>0.05).

### Correlation between expression of HSPs and patient gender and age, occurrence site of tumor, size of tumor, malignant degree and occurrence of neural invasion and metastasis

Table I shows that the expression rates (χ^2^=4.1831 and 7.3758, respectively) and expression strengths (χ^2^=6.579 and 7.516, respectively) of HSP27 and HSP60 were significantly lower in the tumor from the patients aged ≥50 years than those from the patients aged <50 years (each P<0.05). This suggests that the expression strengths of HSP27 and HSP60 were both negatively correlated with patient age (r=−0.365 and −0.404, respectively; each P<0.05).

The positive strengths of HSP70 and HSP86 were weaker in the parotid group, but stronger in the submandibular and minor salivary gland groups (χ^2^= 6.1017, P<0.05 and χ^2^=10.2228 and P<0.01, respectively). There was a significantly positive correlation among the above three groups (r=0.480, P=0.002 and r=0.4822, P<0.01, respectively). In particular, both HSP70 and HSP8 were both significantly stronger in the minor salivary gland group than in the parotid group (χ^2^=9.8873 and 7.2865, respectively; each P<0.01).

The samples were divided into 3 groups based on the diameter of the tumor tissue; <2 cm group, 2–4 cm group and >4 cm group. Using these groupings, the expression rate of HSP27 was significantly lower in the >4 cm group (χ^2^=7.6190, P=0.022), but no difference existed between the <2 cm group and the 2–4 cm group. If the samples were divided into 2 groups based on the diameter of the tumor tissue; <4 cm group and >4 cm group, then a negative correlation between the expression rate of HSP27 and tumor size was observed (χ^2^= 6.8089, P= 0.009, r=−0.4193, P<0.001). Compared with the 2–4 cm group, the expression rate and positive strength of HSP27 were also significantly negatively correlated in the >4 cm group (r=−0.5185 and −0.3790, respectively; each P<0.05).

Compared with the low grading malignant group, the expression strength of HSP86 was significant higher in the middle-high grade malignant group (χ^2^=12.886, P=000), and was positively correlated with malignant grading (r=0.487, P=0.001).

Upon comparing accompanying neural invasion or metastasis, it was revealed that the expression rates of HSP27 significantly decreased (χ^2^=9.3411 and 7.1795, respectively, each P<0.05). The expression strength of HSP27 also decreased with neural invasion or metastasis, (χ^2^=9.655 and 8.169, respectively, each P<0.05) and was negatively correlated with neural invasion and metastasis (r=−0.474, P<0.01 and r=−0.351, P<0.05, respectively).

By contrast, the expression strength of HSP70 (χ^2^=8.749 and 5.914, respectively) and HSP86 (χ^2^=5.192 and 6.852, respectively) increased with neural invasion and metastasis (each P<0.05) and a positive correlation was found between the expression strength and neural invasion (r=0.447, P<0.01; r= 0.355, P<0.05, respectively) or metastasis (r=0.376, P<0.05 and r=0.406, P<0.01, respectively).

### Correlation analysis of HSPs with PI in malignant salivary gland tumors

**Figure 3:**
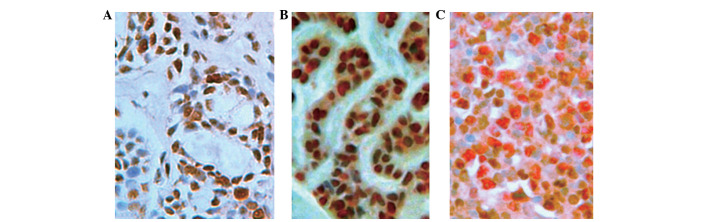
Expression of PCNA and co-expression with HSPs in salivary gland malinant tumors. (A) In adenoid cystic carcinoma, higher PCNA-PI was located in the nucleus of tumor cells. (IHC-SP staining; magnification, ×400). (B) PCNA (AP-Red staining indicated by bright red color) and HSP86 (DAB staining indicated by brownish yellow color) co-located in the nuclei of tumor cells (IHC-Dou SP staining; magnification, ×400). (C) PCNA (AP-Red staining indicated by bright red color) and HSP27 (DAB staining indicated by brownish-yellow color) were respectively located in the nuclei and plasma of same or different tumor cells, with reverse expression trend (IHC-Dou SP staining; magnification, ×400). PCNA, proliferating cell nuclear antigen; HSP, heat shock protein; PCNA-PI, proliferating cell nuclear antigen-proliferating index.

PCNA was mainly located in the nucleus of tumor epithelial cells (Fig. 3A) and little was expressed in non-tumor salivary gland tissues. However, its expression ratio increased in both benign and malignant tumor tissues. In the benign tumor group, the expression rate was 58.54% (24/41) with PI reaching 23.46±33.177, while in the malignant tumor group, it was 100% and 49.95±14.569. Therefore, significant differences existed between the benign group and the malignant group (t=−4.6323, P=0.0000).

**Figure 4:**
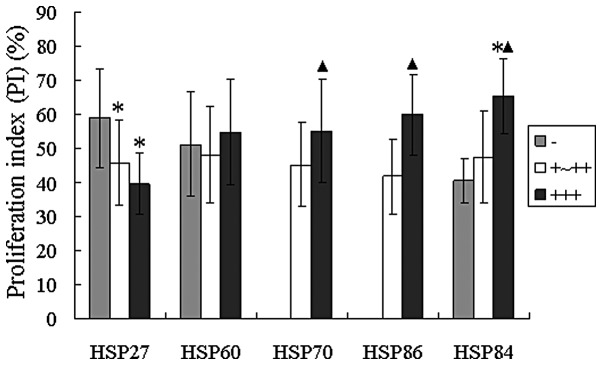
Correlation between the expression of 5 HSPs and PCNA-PI in salivary gland malinant tumors. In salivary gland malinant tumors, the PCNA-PI was positively correlated with expression of HSP70, HSP86 and HSP84 (each P<0.05), but was negatively correlated with HSP27. ^*^Comparison between the group of +/++ or +++ and the − group. ^▴^Comparison between the +/++ group and the +++ group. HSPs, heat shock proteins; PCNA-PI, proliferating cell nuclear antigen-proliferating index.

The correlation between the expression of the 5 HSPs and PI was analyzed in malignant tumors. (Figs. 3B and C and 4) demonstrated that the PCNA-PI was significantly higher (P<0.05), while the expression strength of HSP70, HSP86 and HSP84 was enhanced in the malignant tumors (t=−4.0687, −5.0744 and −3.1779, respectively). Also, PCNA-PI was highest with the lack of HSP27 expression, but HSP27 positive (+/++, +++) PCNA-PI was significantly lower (t=2.9185 and 2.1834, respectively). The above results of duration t-test all had statistical significance (each P<0.05).

## Discussion

In this study, 5 HSPs were found to be expressed in salivary gland tumors (expression rates, 64.71 to 100%) and non-tumor salivary ducts, but not in salivary glands. The expression of the 5 HSPs was closely correlated with the occurrence of salivary gland tumors originating from salivary ducts, but that varied with the tumor type.

HSP27 is a member of the small HSP family, and is associated with tumor formation and metastatic potential (9,10). It may be a potential target for the periodontal regeneration process involved in cell migration (11). Our findings indicated that the significant decrease in expression rate and strength in malignant tumors was similar to that of low class gliomas (12). Thus, HSP27 may be correlated with cell differentiation of salivary gland tumors, and its weak expression may be used as a marker of low differentiation in malignant tumors. Due to the growth rate, the ability to invade and metastasize was often stronger in the low differentiated tumors, so the expression of HSP27 significantly decreased in both the >4 cm diameter group and groups where neural invasion or metastasis occurred (each P<0.05). Therefore, low or no expression of HSP27 may be a useful biomarker to evaluate the biological behavior of malignant salivary gland tumors.

HSP60 (also known as Cpn60) is a chaperonin protein, which plays an important role in cell physiology and survival, and is closely correlated with functions of the innate and adaptive immune system in mammals (13). Thus, it is essential in tumor immunity, and is a good biomarker for assessment of prognosis of patients with lung adenocarcinoma (14,15). We found that the expression of HSP60 was negatively correlated with the age of patients; this suggests that as an immune adjuvant, the age of the patients is a negative control factor. Its decreasing expression may lead to a decline in antitumor immune function and may be correlated with occurrence of metastasis. HSP60 may participate in tumor immune action in the early stage of tumor development. Similarly, age factors may also be an important factor associated with HSP27 expression. However, the exact underlying mechanisms for the effect of the patient age on the expression of HSP60 and HSP27 remain unclear.

By far the most conserved molecular chaperone, HSP70 is an important apoptosis inhibitor (16) and is often increasingly synthesized in malignant tumors (17,18), including hepatocellular carcinoma HepG (2) cells. When the HSP70 gene was silenced, cell death induced by chemotherapy drugs was enhanced (19).

HSP86 (90α) and HSP84 (90β) are two subtypes of HSP90, which are functionally important in the structure and stability of client proteins in tumor cells, which affect proliferation, survival, differentiation, mobility, angiogenesis and metastasis of tumor cells (20,21). Hop (Hsp70/Hsp90 organising protein), also called stress-inducible protein 1, which interacts with HSP70 and HSP90 was overexpressed in human colonic carcinoma (22), invasive pancreatic cancer cell lines and malignant tissues of pancreatic cancer patients, and knockdown of Hop gene may decrease the invasiveness of pancreatic cancer cells, possibly by means of modulation of HSP90 activity (23).

Our results demonstrated that HSP70, HSP86 and HSP84 were all increasingly expressed in salivary gland tumors and were positively correlated with malignant tumors (r= 0.360, 0.457 and 0.290, respectively); particularly for HSP86 (r= 0.487). The expression of HSP70 and HSP86 were also positively correlated with the occurrence of neural invasion (r= 0.447 and 0.355, respectively) and metastasis (r= 0.376 and 0.406, respectively), as demonstrated in previous studies (18,21,22). These findings suggest that HSP70 and HSP90 and the client proteins synthesized by the lasting induction of HSP70 and HSP90 work together to enhance the occurrence and development of malignant tumors. Thus, in increasing order, the expression of HSP70 and HSP86 was arranged as parotid gland, submandibular and minor salivary glands (r=0.480 and 0.482, respectively).

In 41 cases of benign tumors, expression of HSP84 and HSP86 was significantly greater in mixed tumors, particularly when compared with that in the adenolymphoma group. This may be due to the higher recurrence and malignant transformation rates in mixed tumor compared to adenoma and adenolymphoma. As previously reported (22), HSP90 may be a critical molecular chaperone in tumor recurrence and malignant transformation.

PCNA is a nucleoprotein that is widely expressed during S phase of the cell cycle and is associated with cell proliferation potential (24); therefore, it is often found at the front of invasive tumors and correlated with poor prognosis (25).

PCNA-PI was mainly found in the tumor cells and increased with HSP70, HSP86 and HSP84 expression (each P<0.05). Moreover, significantly higher levels of PCNA-PI were observed in malignant than in the benign tumors (t=−4.6323, P=0.0000). This indicated that the malignant tumor cells have a stronger proliferative potentiality and this may explain why more HSP70, HSP86 and HSP84 were synthesized. As important molecular chaperones, these HSPs maintained the high proliferation capabilities of the tumor cells, particularly that of the malignant tumors, through interaction with their client proteins. This may explain why the double staining results revealed the higher co-expression correlation between PCNA and HSP70, HSP86 and HSP84.

Salinthone *et al*(26) reported that HSP27 may inhibit cell proliferation. The present study also demonstrated that high expression of HSP27 prevented the proliferation and immortalization of tumor cells in benign tumors and although its expression was weakened, the proliferation and malignant transformation rate of the tumor cell increased. Therefore, PCNA-PI was higher in the group where HSP27 expression was negative (t=2.9185 and 2.1834, respectively; each P<0.05). The reversely proportional correlation identified between HSP27 expression and PCNA based on the double staining results may indicate that HSP27 only has an incomplete inhibition effect on the expression of PCNA in tumor cells and certain other unknown pathways may be involved. The decrease in the expression of HSP27 and positive PCNA may work together to control tumor proliferation and the malignant transformation process.

## Figures and Tables

**Table I:** Correlation between expression of HSPs and patient gender and age, occurrence site of tumor, size of tumor, malignant degree and occurrence of neural invasion and metastasis (n=40).

		Expression of HSPs														
		HSP27			HSP60			HSP70			HSP86			HSP84		
Group	n	−	+/++	+++	−	+/++	+++	−	+/++	+++	−	+/++	+++	−	+/++	+++
Gender																
Male	20	7	11	2	6	12	9	0	14	9	0	11	9	2	13	5
Female	20	7	12	1	8	10	2	0	10	10	0	11	9	2	16	2
Age (years)		r=−0.365 P=0.021^a^			r=−0.404 P=0.010^a^											
<50	23	5	15	3	4	16	3	0	11	12	0	13	10	2	16	5
≥50	17	9	8	0	10	6	1	0	10	7	0	9	8	2	13	2
Location									r=0.480 P=0.002^b^		r=0.482 P=0.002^b^					
Parotid gland	26	8	15	3	11	13	2	0	18	8	0	19	7	4	19	3
Submandibular gland	5	1	4	0	2	2	1	0	2	3	0	1	4	0	4	1
Minor salivary gland	9	5	4	0	1	7	1	0	1	8	0	2	7	0	6	3
Size (cm)		χ^2^=10.660 P=0.031 ^a^														
≤2	12	4	8	0	5	6	1	0	5	7	0	7	5	1	9	2
2–4	20	4	13	3	4	14	2	0	13	7	0	11	9	1	16	3
>4	8	6	2	0	5	2	1	0	3	5	0	4	4	2	4	2
Grading											r=0.487 P=0.001 ^b^					
Low	9	2	6	1	4	5	0	0	7	2	0	9	0	1	8	0
Middle-high	31	12	17	2	10	17	4	0	14	17	0	13	18	3	21	7
Neural invasion		r=−0.474 P=0.002 ^b^						r=0.447 P=0.004^b^			r=0.355 P=0.025 ^a^					
−	31	7	21	3	11	17	3	0	20	11	0	20	11	3	23	5
+	9	7	2	0	3	5	1	0	1	8	0	2	7	1	6	2
Metastasis		r=−0.351 P=0.026^a^			χ^2^=6.916 P=0.031^a^			r=0.376 P=0.017^a^			r=0.406 P=0.009 ^b^					
−	30	7	21	2	8	20	2	0	19	11	0	20	10	3	24	3
+	10	7	2	1	6	2	2	0	2	8	0	2	8	1	5	4

a P<0.05;

b P<0.01. HSPs, heat shock proteins.
